# systemPipeR: NGS workflow and report generation environment

**DOI:** 10.1186/s12859-016-1241-0

**Published:** 2016-09-20

**Authors:** Tyler W. H. Backman, Thomas Girke

**Affiliations:** Institute for Integrative Genome Biology, University of California, Riverside, 1207F Genomics Building, 3401 Watkins Drive, Riverside, 92521 CA, USA

**Keywords:** Analysis workflow, Next Generation Sequencing (NGS), Ribo-Seq, ChIP-Seq, RNA-Seq, VAR-Seq

## Abstract

**Background:**

Next-generation sequencing (NGS) has revolutionized how research is carried out in many areas of biology and medicine. However, the analysis of NGS data remains a major obstacle to the efficient utilization of the technology, as it requires complex multi-step processing of big data demanding considerable computational expertise from users. While substantial effort has been invested on the development of software dedicated to the individual analysis steps of NGS experiments, insufficient resources are currently available for integrating the individual software components within the widely used R/Bioconductor environment into automated workflows capable of running the analysis of most types of NGS applications from start-to-finish in a time-efficient and reproducible manner.

**Results:**

To address this need, we have developed the R/Bioconductor package *systemPipeR*. It is an extensible environment for both building and running end-to-end analysis workflows with automated report generation for a wide range of NGS applications. Its unique features include a uniform workflow interface across different NGS applications, automated report generation, and support for running both R and command-line software on local computers and computer clusters. A flexible sample annotation infrastructure efficiently handles complex sample sets and experimental designs. To simplify the analysis of widely used NGS applications, the package provides pre-configured workflows and reporting templates for RNA-Seq, ChIP-Seq, VAR-Seq and Ribo-Seq. Additional workflow templates will be provided in the future.

**Conclusions:**

*systemPipeR* accelerates the extraction of reproducible analysis results from NGS experiments. By combining the capabilities of many R/Bioconductor and command-line tools, it makes efficient use of existing software resources without limiting the user to a set of predefined methods or environments. *systemPipeR* is freely available for all common operating systems from Bioconductor (http://bioconductor.org/packages/devel/systemPipeR).

**Electronic supplementary material:**

The online version of this article (doi:10.1186/s12859-016-1241-0) contains supplementary material, which is available to authorized users.

## Background

By allowing scientists to rapidly sequence and quantify DNA and RNA molecules, next-generation sequencing (NGS) technology has transformed biology into one of the most data intensive research disciplines. In the past, experiments have been performed on a gene-by-gene basis, while NGS has introduced an age where it is has become a routine to sequence entire transcriptomes, genomes or epigenomes rather than their isolated parts of interest. It will soon be possible to conduct these experiments on large numbers of single cell samples [1, 2] for a wide range of time points, treatments, and genetic backgrounds to study biological systems with greater resolution and precision. Sequencing the genetic material of each individual within entire populations of organisms of the same species or genus will enable the study of adaptation processes [3], disease progression, and micro-evolution in real time [4]. This technological shift empowers researchers to address questions at a genome-wide scale, for example by profiling the mRNA, miRNA and DNA methylation states of a large set of biological samples in parallel [5].

The success of NGS-driven research has led to a data explosion of increasing size and complexity, making it now more time consuming and challenging for researchers to extract knowledge from their experiments. Rapid processing of the results is essential to test, refine, and formulate new hypotheses for designing follow-up experiments. As a result, biologists have to dedicate nowadays substantial time to data analysis tasks while training themselves effectively as genome data scientists rather than focusing on experimentation as they used to in the past.

In recent years, a considerable number of algorithms, statistical methods, and software tools has been developed to perform the individual analysis steps of different NGS applications. These include short read pre-processors, aligners, variant and peak callers, as well as statistical methods for the analysis of genomic regions that are differentially expressed [6, 7], bound [8] or methylated [9, 10]. Also essential are tools for processing short read alignments [11], genomic intervals [12, 13] and annotations [14]. However, most data analysis routines of NGS applications are very complex involving multiple software tools for their many processing steps. As a result, there is a great need for flexible software environments connecting the individual software components to automated workflows in order to perform complex genome-wide analyses in an efficient and reproducible manner. While many workflow management resources exist [15–24] for a variety of data analysis programming languages (for details see below), only insufficient general purpose NGS workflow solutions are currently available for the popular R programming language. R and the affiliated Bioconductor environment provide a substantial number of widely used tools with a large user base in this area [10]. Thus, a workflow framework for federating NGS applications from within R will have many benefits for experimental and computational scientists who use R for NGS data analysis.

To address this need, we designed *systemPipeR* as a Bioconductor package for building and running workflows for most NGS applications with support for integrating a wide array of command-line and R/Bioconductor software.

## Implementation

### Environment

*systemPipeR* has been implemented as an open-source Bioconductor package using the R programming language for statistical computing and graphics. R was chosen as the core development platform for *systemPipeR* because of the following reasons. (i) R is currently one of the most popular statistical data analysis and programming environments in bioinformatics. (ii) Its external language bindings support the implementation of computationally time-consuming analysis steps in high-performance languages such as C/C++. (iii) It supports advanced parallel computation on multi-core machines and computer clusters. (iv) A well developed infrastructure interfaces R with several other popular programing languages such as Python. (v) R provides advanced graphical and visualization utilities for scientific computing. (vi) It offers access to a vast landscape of statistical and machine learning tools. (vii) Its integration with the Bioconductor project promotes reusability of genomics software components, while also making efficient use of a large number of existing NGS packages that are well tested and widely used by the community. To support long-term reproducibility of analysis outcomes, *systemPipeR* is also distributed as Docker image of Bioconductor’s sequencing division. Docker containers provide an efficient solution for packaging complex software together with all its system dependencies to ensure it will run the same in the future across different environments, including different operating systems and cloud-based solutions.

### Workflow design

**Fig. 1:**
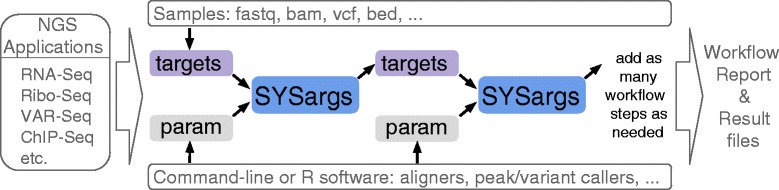
Workflow steps with input/output file operations are controlled by *SYSargs* objects. Each *SYSargs* instance is constructed from a *targets* and a *param* file. The only input required from the user is the initial *targets* file. Subsequent instances are created automatically. Any number of predefined or custom workflow steps is supported

*systemPipeR* workflows (Fig. 1) can be run from start-to-finish with a single command, or stepwise in interactive mode from the R console. New workflows are constructed, or existing ones modified, by connecting so-called *SYSargs* workflow control modules (R S4 class). Each *SYSargs* module contains instructions needed for processing a set of input files with a specific command-line or R software; as well as the paths to the corresponding outputs generated by a specific NGS tool such as a read preprocessor (trimmed/filtered FASTQ files), aligner (SAM/BAM files), read counter, variant caller (VCF/BCF files), peak caller (BED/WIG files) or statistical function. Typically, the only input the user needs to provide for running workflows is a single tabular *targets* file containing the paths to the initial sample input files (e.g. FASTQ) along with sample labels, and if appropriate biological replicate and contrast information for controlling differential abundance analyses (e.g. gene expression). Downstream derivatives of these *targets* files along with the corresponding *SYSargs* instances (see Fig. 1) are created automatically within each workflow. The parameters required for running command-line software are provided by parameter (*param*) files described below. For R-based workflow steps, *param* files are not required but can be useful for operations importing and/or exporting sample-level files. This modular design has several advantages. First, it provides a high level of flexibility for designing workflows, such as allowing the user to start workflows from the very beginning or anywhere in-between (e.g. FASTQ or BAM level). Second, it is straightforward to add custom workflow steps without requiring computational expert knowledge from users. Workflows can also have any number of steps including branch points. Lastly, it also minimizes errors as all input and output files are registered, and sample labels specified in the initial targets file will be consistently used throughout all workflow results including plots, tables and workflow reports.

### Command-line software support

An important feature of *systemPipeR* is support for running command-line software directly from R on both single machines or computer clusters. This offers several advantages such as seamless integration of most command-line software available in the NGS field with the extensive genome analysis resources provided by R/Bioconductor. The user interface for running command-line software has been generalized as a single function for ease of use, while only one additional command will run the same tool in parallel mode on a computer cluster (see below). Examples of command-line software used by *systemPipeR*’s preconfigured workflow templates (see below) include the aligners *BWA-MEM* [25], *Bowtie2* [26], *TopHat2* [27], *HISAT2* [28], as well as the peak/variant callers *MACS* [29], *GATK* [30] and *BCFtools* [11]. Support for additional command-line NGS software can be added by simply providing the argument settings of a chosen software in a tabular *param* file. If appropriate, new *param* files can be permanently included in the package to share them with the community. Functionality for creating *param* files automatically will be provided in the future. This will allow users to create new *param* instances simply by providing an example of the command-line syntax of a chosen software tool. Major advantages of running command-line software from within *systemPipeR* include: a uniform sample management infrastructure within and across workflows; integration of *BatchJobs’* [31] efficient error managment infrastructure for job submissions on computer clusters; the simplicity of restarting failed processes; as well as seamless addition of new samples (e.g. FASTQ or BAM files). In case of a restart, the system will skip the analysis steps of already completed samples and only perform the analysis of the missing ones. If required, any workflow step can be rerun on demand for all or a subset of samples. When submitting command-line software to computer clusters, *BatchJobs* monitors the status of job submissions and alerts users of exceptions, while recording warning and error messages for each process in a log directory with a database-like structure that is accessible from within R or the command-line. This organization helps to diagnose and resolve errors.

### Parallel evaluation

The processing time for NGS experiments can be greatly reduced by making use of parallel evaluation across several CPU cores on single machines, or multiple nodes of computer clusters and cloud-based systems. *systemPipeR* simplifies these parallelization tasks without creating any limitations for users who do not have access to high-performance computer (HPC) resources by providing the option to run workflows in serial or parallel mode. The parallelization functionalities available in *systemPipeR* are largely based on existing and well maintained R packages, mainly *BatchJobs* and *BiocParallel* [31]. By making use of cluster template files, most schedulers and queueing systems are also supported (e.g. Torque, Sun Grid Engine, Slurm). If required, entire workflows can be executed in parallel mode by issuing a single command, while simultaneously generating a detailed analysis report (for details see below). If sufficient parallel computer resources are available, *systemPipeR* can complete the entire analysis workflow of several complex NGS experiments each containing large numbers of FASTQ files within hours rather than days or weeks as can be the case for non-parallelized workflows.

### Automated analysis reports

*systemPipeR* generates automated analysis reports with *knitr* and *R markdown* [32]. These modern reporting environments integrate R code with LaTeX or Markdown. During the evaluation of the R code, reports are dynamically generated in PDF or HTML format. A caching system allows to re-execute selected workflow reporting steps without repeating unnecessary components. This way one can generate reports that resemble a research paper where user generated text is combined with analysis results. This includes support for citations, autogenerated bibliographies, code chunks with syntax highlighting and inline evaluation of variables to update text content. Data components in a report such as tables and figures are updated automatically when rebuilding the document and/or rerunning workflows partially or entirely.

## Results and discussion

### Overview

**Fig. 2:**
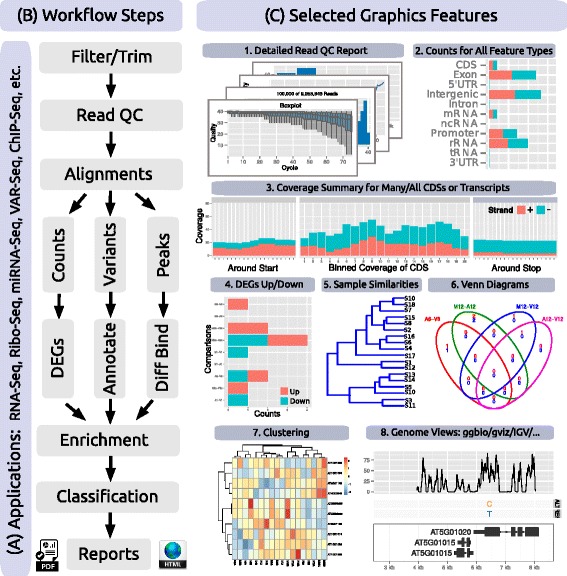
Workflow Steps and Graphical Features. Relevant workflow steps of several NGS applications (**a**) are illustrated in form of a simplified flowchart (**b**). Examples of *systemPipeR’s* functionalities are given under (**c**) including: (1) eight different plots for summarizing the quality and diversity of short reads provided as FASTQ files; (2) strand-specific read count summaries for all feature types provided by a genome annotation; (3) summary plots of read depth coverage for any number of transcripts with nucleotide resolution upstream/downstream of their start and stop codons, as well as binned coverage for their coding regions; (4) enumeration of up- and down-regulated DEGs for user defined sample comparisons; (5) similarity clustering of sample profiles; (6) 2-5-way Venn diagrams for DEGs, peak and variant sets; (7) gene-wise clustering with a wide range of algorithms; and (8) support for plotting read pileups and variants in the context of genome annotations along with genome browser support

*systemPipeR* provides utilities for building and running NGS analysis workflows. To adapt to community standards, widely used R/Bioconductor packages are integrated where possible. This includes the Bioconductor packages *ShortRead*, *Biostrings* and *Rsamtools* for processing sequence and alignment files [33]; *GenomicRanges*, *GenomicAlignments* and *GenomicFeatures* for handling genomic range operations, read counting and annotation data [12]; *edgeR* and *DESeq2* for differential abundance analysis [6, 7]; and *VariantTools* and *VariantAnnotation* for filtering and annotating genome variants [34]. If necessary, one can substitute most of these packages with alternative R or command-line tools. Because many NGS applications share overlapping analysis needs (Fig. 2a), certain workflow steps are conceptualized in *systemPipeR* by a single generic function with support for application-specific parameter settings (Table 1). For instance, most NGS applications involve a short read alignment step (see Fig. 2b), but with very distinct mapping requirements, such as splice junction awareness and variant tolerance for RNA-Seq and VAR-Seq, respectively. To simplify their execution for the user, the different aligners can be run with the same runCommandline function where the software and its parameter settings are specified in the corresponding *SYSargs* instance (see above and Fig. 1).

**Table 1:** Selected functions. The table lists a subset of over 50 methods and functions defined by *systemPipeR*. Usage instructions are provided in the corresponding help pages and vignettes of the package

Function name	Description
genWorkenvir	Generates workflow templates provided by *systemPipeRdata* helper package
systemArgs	Constructs SYSargs workflow control module (S4 object) from*targets* and *param* files
runCommandline	Executes command-line software on samples and parameters specified in SYSargs
clusterRun	Runs command-line software in parallel mode on a computer cluster
preprocessReads	Filtering and/or trimming of short reads using predefined or custom parameters
seeFASTQ/seeFASTQplot	Generates quality reports for any number of FASTQ files
alignStats	Generates alignment statistics, such as total number of reads and alignment frequency
run_edgeR/run_DESeq2	Runs *edgeR* or *DESeq2* for any number of pairwise sample comparisons
filterDEGs	Filters and plots DEG results based on user-defined parameters
overLapper/vennPlot	Computation of Venn intersects for 2-20 or more samples and 2-5 way Venn diagrams
GOCluster_Report	GO term enrichment analysis for large numbers of gene sets
variantReport	Generates a variant report containing genomic annotations and confidence statistics
predORF	Prediction of short open reading frames in DNA sequences
featuretypeCounts	Computes and plots read distribution for many feature types at once
featureCoverage	Computes and plots read depth coverage from many transcripts

### Workflow templates

*systemPipeR* also provides end-to-end workflow templates for RNA-Seq, Ribo-Seq, ChIP-Seq and VAR-Seq analysis. A detailed vignette (manual) is provided for each workflow, while an overview vignette introduces the general design concepts. Templates for additional NGS applications will be made available in the future. To test workflows quickly or design new ones from existing templates, users can generate with a single command (genWorkenvir) workflow instances fully populated with sample data and parameter files required for running a chosen workflow. The corresponding sample data are provided by the affiliated data package *systemPipeRdata*, also available from Bioconductor. To illustrates the utilities of *systemPipeR’s* workflow templates, a case study has been included as Additional file 1 that guides the reader through the most important steps of a sample workflow. A typical gene-level RNA-Seq analysis was chosen here because it is currently one of the most widely used applications in the NGS field.

### Add-on tools

In addition to providing a framework for running NGS analysis workflows, *systemPipeR* includes many functions and methods that expand and enhance its workflows. The following gives selected examples of these utilities (also illustrated in Fig. 2c and Table 1). A read pre-processor function (preprocessReads) addresses the often very sophisticated quality filtering and adaptor trimming needs of specialized NGS applications such as Ribo-Seq or smallRNA-Seq. The functions seeFastq and seeFastqPlot generate and plot detailed quality reports for FASTQ files (Fig. 2c1). These reports are easy to generate and designed to facilitate the visual inspection of large numbers of FASTQ files in a single report. The featuretypeCounts function computes and plots the distribution of reads across all features available in a genome annotation rather than just a single one (Fig. 2c2). The featureCoverage function generates from genome-level alignments read depth coverage summaries for all or a subset of transcripts with nucleotide resolution upstream/downstream of their start and stop codons, as well as binned coverage for their coding regions (Fig. 2c3). Additional utilities include functions to automate the analysis of differentially expressed genes (DEGs) with *edgeR* or *DESeq2* (Fig. 2c4), to compute Venn intersects for large numbers of sample sets (e.g. 2-20 or as many as available memory allows) with plotting functionalities for 2-5 way Venn diagrams (Fig. 2c6), and to run gene set enrichment analyses in batch mode on large numbers of gene sets. The modular design of the *systemPipeR* environment allows users to easily substitute any of these built-in tools with alternative R-based or command-line software, such as using FastQC [35], FASTX-Toolkit [36] or MultiQC [37] for quality reporting, read trimming or result aggregation, respectively.

### Performance and scalability

*systemPipeR* has been optimized to run workflows in a time and memory efficient manner even on very large read sets from complex genomes (e.g. mammalian genomes). This is achieved by making heavy use of indexing, file streaming and parallelization functionalities. For instance, users can limit the RAM requirements of several workflow steps by specifying the maximum number of reads or alignments to stream into memory at any time. This enables analysis of very large files with tens of GBs of storage space on systems with limited RAM resources, making it possible to run *systemPipeR* workflows even on laptops or smaller workstations, provided they have the required software installed and enough disk space available for storing large NGS input and result files. The processing time of non-parallelized analysis steps depends on the time performance of a specific software tool chosen for a workflow step. For instance, in the RNA-Seq workflow described under Additional file 1 the alignment step will run on a single sample (FASTQ file) with the native time performance of the chosen aligner *Bowtie2/Tophat2*. Using the much faster *HISAT2* aligner instead would accelerate the alignment step proportionally to the time improvements provided by this aligner without the need of additional parallel computer resources [28]. On a computer cluster, parallelized *systemPipeR* workflows scale nearly linearly in time with the number of sample files (i.e. FASTQ files) since every step can be parallelized at the sample level. In practice this means, the runtime of an analysis of 100 FASTQ files can be accelerated by 10 or 100 fold when using instead of a single CPU core 10 or 100 CPU cores, respectively. For example, the RNA-Seq workflow in Additional file 1 can process 100 FASTQ files, each with 30–40 million reads from a mammalian genome, in 6–8 hours using 100 CPU cores (CPU Model: AMD 6376, 2.3 GHz) and a maximum RAM requirement of less than 10 GB per node. Since the alignment step with *Bowtie2/Tophat2* accounts for most of the compute time of the entire workflow, the use of faster RNA-Seq aligners, such as *Rsubread* or *HISAT2*, can reduce the compute time to less than 3 hours. With comparable parallel computer resources available, one can complete with *systemPipeR* the end-to-end analysis of several complex NGS experiments each containing 50–100 FASTQ files in less than a day rather than many days or weeks as is common in non-parallelized workflows.

### Need for an R-based NGS workflow environment

Several related software tools with NGS workflow functionality are available. These include *Galaxy* [15, 38], *Snakemake* [16], *Taverna* [17], *BioBlend* [39], *bcbio-nextgen* [18], *Knime* [19], *Ruffus* [20], *Kepler* [21], Wasp [22], *ViennaNGS* [23], *Mercury* [24], *RAP* [40], and *LONI* [41] among others. Additionally, general purpose utilities for workflow management and design are provided by *Rabix* [42] and *WDL* [43]. These tools provide infrastructure for streamlining the analysis of NGS data in a variety of data analysis environments and computer languages. However, only limited resources are available for designing and running analysis workflows for a wide range of NGS applications directly from within R as is possible with *systemPipeR*. One of the few exceptions is *QuasR* [44]. This Bioconductor package supports the initial analysis steps of several NGS applications, but it lacks an interface to integrate external command-line software and functionalities to build new workflows. Other existing R/Bioconductor resources for analyzing NGS data address the needs in this area only partially. For instance, many of them are limited to certain NGS applications, or cover only a subset of the processing steps required for complete workflows; do not support command-line software; or lack workflow design functionalities for different NGS applications. *systemPipeR* has been designed to address these requirements. However, it is important to mention here that well established community workflow environments like *Galaxy* provide several additional features not available in *systemPipeR*. A small subselection of them includes: (i) a web interface to support non-expert users who are not familiar with data analysis programming environments like R; (ii) support for a wider range of data types outside of the NGS field; (iii) a well-established infrastructure and community for archiving and sharing workflow protocols; or (iv) support for additional reporting technologies such as iPython notebooks. To take advantage of this powerful infrastructure, *Galaxy* compatible versions of *systemPipeR*’s NGS workflows will be released in the future. This will allow biologists to run them from an easy-to-use web interface, while also being able to access additional functionalities available in *Galaxy’s* large ecosystem of analysis tools.

## Conclusion

The *systemPipeR* package unites R/Bioconductor resources with external command-line software to standardize and automate the analysis of a wide range of NGS applications. Its functionalities reduce the complexity and time required to translate NGS data into interpretable research results, while a built-in reporting feature improves reproducibility. The environment provides sufficient flexibility to choose the optimal software for each step in complex NGS workflows, customize workflows, and design new workflows. Pre-configured workflow templates are included for several NGS applications. Templates for additional NGS applications are under development and will be added to the package in the near future.

## Availability and requirements

- **Project name:***systemPipeR* workflow environment
- **Project home page:** http://bioconductor.org/
  packages/systemPipeR
- **Archived version:***systemPipeR*
- **Operating system(s):** Platform independent
- **Programming language:** R
- **Other requirements:** R version ≥3.2, Bioconductor version ≥3.2
- **License:** Artistic-2-0
- **Any restrictions to use by non-academics:** none

## Additional file

Additional file 1 RNA-Seq Workflow Example. Case study to illustrate the utilities of *systemPipeR* using an RNA-Seq workflow as example. (PDF 89 kb)

## Abbreviations

BAM: Binary version of sequence alignment map format
ChIP-Seq: Chromatin immunoprecipitation sequencing
DEG: Differentially expressed genes
FASTQ: short read sequence file format
NGS: Next generation sequencing
Ribo-Seq: NGS profiling of mRNA populations bound to ribosomes
RNA-Seq: NGS profiling of mRNA
SAM: Sequence alignment map format
VAR-Seq: NGS-based variant detection

## References

[1] Kalisky T, Quake SR (2011). Single-cell genomics. Nat Methods.

[2] Trapnell C, Cacchiarelli D, Grimsby J, Pokharel P, Li S, Morse M, Lennon NJ, Livak KJ, Mikkelsen TS, Rinn JL (2014). The dynamics and regulators of cell fate decisions are revealed by pseudotemporal ordering of single cells. Nat Biotechnol..

[3] Lindblad-Toh K, Garber M, Zuk O, Lin MF, Parker BJ, Washietl S, Kheradpour P, Ernst J, Jordan G, Mauceli E, Ward LD, Lowe CB, Holloway AK, Clamp M, Gnerre S, Alföldi J, Beal K, Chang J, Clawson H, Cuff J, Di Palma F, Fitzgerald S, Flicek P, Guttman M, Hubisz MJ, Jaffe DB, Jungreis I, Kent WJ, Kostka D, Lara M, Martins AL, Massingham T, Moltke I, Raney BJ, Rasmussen MD, Robinson J, Stark A, Vilella AJ, Wen J, Xie X, Zody MC, Baldwin J, Bloom T, Chin CW, Heiman D, Nicol R, Nusbaum C, Young S, Wilkinson J, Worley KC, Kovar CL, Muzny DM, Gibbs RA, Cree A, Dihn HH, Fowler G, Jhangiani S, Joshi V, Lee S, Lewis LR, Nazareth LV, Okwuonu G, Santibanez J, Warren WC, Mardis ER, Weinstock GM, Wilson RK, Delehaunty K, Dooling D, Fronik C, Fulton L, Fulton B, Graves T, Minx P, Sodergren E, Birney E, Margulies EH, Herrero J, Green ED, Haussler D, Siepel A, Goldman N, Pollard KS, Pedersen JS, Lander ES, Kellis M, Broad Institute Sequencing Platform and Whole Genome Assembly Team, Baylor College of Medicine Human Genome Sequencing Center Sequencing Team, Genome Institute at Washington University (2011). A high-resolution map of human evolutionary constraint using 29 mammals. Nature.

[4] Kato-Maeda M, Ho C, Passarelli B, Banaei N, Grinsdale J, Flores L, Anderson J, Murray M, Rose G, Kawamura LM, Pourmand N, Tariq MA, Gagneux S, Hopewell PC (2013). Use of whole genome sequencing to determine the microevolution of *Mycobacterium tuberculosis* during an outbreak. PLoS ONE.

[5] Holt RA, Jones SJ (2008). The new paradigm of flow cell sequencing. Genome Res.

[6] Robinson MD, McCarthy DJ, Smyth GK (2010). edgeR: a Bioconductor package for differential expression analysis of digital gene expression data. Bioinformatics.

[7] Love M, Huber W, Anders S (2014). Moderated estimation of fold change and dispersion for RNA-seq data with DESeq2. Genome Biol.

[8] Kharchenko PV, Tolstorukov MY, Park PJ (2008). Design and analysis of ChIP-seq experiments for DNA-binding proteins. Nat Biotechnol.

[9] Akalin A, Kormaksson M, Li S, Garrett-Bakelman FE, Figueroa ME, Ari M, Mason CE (2012). methylKit: a comprehensive R package for the analysis of genome-wide DNA methylation profiles. Genome Biol.

[10] Huber W, Carey VJ, Gentleman R, Anders S, Carlson M, Carvalho BS, Bravo HC, Davis S, Gatto L, Girke T, Gottardo R, Hahne F, Hansen KD, Irizarry RA, Lawrence M, Love MI, MacDonald J, Valerie O, Oleś AK, Pagès H, Reyes A, Shannon P, Smyth GK, Tenenbaum D, Waldron L, Morgan M (2015). Orchestrating high-throughput genomic analysis with Bioconductor. Nat Methods.

[11] Li H, Handsaker B, Wysoker A, Fennell T, Ruan J, Homer N, Marth G, Abecasis G, Durbin R, 1000 Genome Project Data Processing Subgroup (2009). The Sequence Alignment/Map format and SAMtools. Bioinformatics.

[12] Lawrence M, Huber W, Pagès H, Aboyoun P, Carlson M, Gentleman R, Morgan MT, Carey VJ (2013). Software for computing and annotating genomic ranges. PLoS Comput Biol.

[13] Quinlan AR, Hall IM (2010). BEDTools: a flexible suite of utilities for comparing genomic features. Bioinformatics.

[14] Durinck S, Moreau Y, Kasprzyk A, Davis S, De Moor B, Brazma A, Wolfgang H (2005). BioMart and Bioconductor: a powerful link between biological databases and microarray data analysis. Bioinformatics.

[15] Goecks J, Nekrutenko A, Taylor J, Galaxy Team (2010). Galaxy: a comprehensive approach for supporting accessible reproducible, and transparent computational research in the life sciences. Genome Biol.

[16] Köster J, Rahmann S (2012). Snakemake–a scalable bioinformatics workflow engine. Bioinformatics.

[17] Wolstencroft K, Haines R, Fellows D, Williams A, Withers D, Owen S, Soiland-Reyes S, Dunlop I, Nenadic A, Fisher P, Bhagat J, Belhajjame K, Bacall F, Hardisty A, Nieva de la Hidalga A, Balcazar Vargas MP, Sufi S, Goble C (2013). The taverna workflow suite: designing and executing workflows of web services on the desktop, web or in the cloud. Nucleic Acids Res.

[18] Guimera RV (2012). bcbio-nextgen: Automated, distributed next-gen sequencing pipeline. EMBnet J.

[19] Warr WA (2012). Scientific workflow systems: Pipeline pilot and KNIME. J Comput Aided Mol Des.

[20] Goodstadt L (2010). Ruffus: a lightweight python library for computational pipelines. Bioinformatics.

[21] Stropp T, McPhillips T, Ludäscher B, Bieda M (2012). Workflows for microarray data processing in the kepler environment. BMC Bioinformatics.

[22] McLellan AS, Dubin RA, Jing Q, Broin PO, Moskowitz D, Suzuki M, Calder RB, Hargitai J, Golden A, Greally JM (2012). The wasp system: an open source environment for managing and analyzing genomic data. Genomics.

[23] Wolfinger MT, Fallmann J, Florian E, Amman F (2015). ViennaNGS: A toolbox for building efficient next- generation sequencing analysis pipelines. F1000Res.

[24] Reid JG, Carroll A, Narayanan V, Dahdouli M, Sundquist A, English A, Bainbridge M, White S, Salerno W, Buhay C, Yu F, Donna M, Daly R, Duyk G, Gibbs RA, Boerwinkle E (2014). Launching genomics into the cloud: deployment of Mercury, a next generation sequence analysis pipeline. BMC Bioinformatics.

[25] Li H. Aligning sequence reads, clone sequences and assembly contigs with BWA-MEM. 2013. arXiv:1303.3997v2. http://arxiv.org/abs/1303.3997v2.

[26] Langmead B, Salzberg SL (2012). Fast gapped-read alignment with Bowtie 2. Nat Methods.

[27] Kim D, Pertea G, Trapnell C, Pimentel H, Kelley R, Salzberg SL (2013). TopHat2: accurate alignment of transcriptomes in the presence of insertions, deletions and gene fusions. Genome Biol..

[28] Kim D, Langmead B, Salzberg SL (2015). HISAT: a fast spliced aligner with low memory requirements. Nat Methods.

[29] Zhang Y, Liu T, Meyer CA, Eeckhoute J, Johnson DS, Bernstein BE, Nussbaum C, Myers RM, Brown M, Li W, Liu XS (2008). Model-based analysis of ChIP-Seq (MACS). Genome Biol.

[30] McKenna A, Hanna M, Banks E, Sivachenko A, Cibulskis K, Andrew K, Garimella K, Altshuler D, Stacey G, Daly M, DePristo MA (2010). The Genome Analysis Toolkit: a MapReduce framework for analyzing next-generation DNA sequencing data. Genome Res.

[31] Bischl B, Lang M, Mersmann O, Rahnenführer J, Weihs C (2015). BatchJobs and BatchExperiments: abstraction mechanisms for using R in batch environments. J Stat Softw.

[32] Xie Y (2013). Dynamic Documents with R and Knitr (Chapman & Hall/CRC The R Series).

[33] Morgan M, Anders S, Lawrence M, Aboyoun P, Pagès H, Gentleman R (2009). ShortRead: a Bioconductor package for input, quality assessment and exploration of high throughput sequence data. Bioinformatics.

[34] Obenchain V, Lawrence M, Carey V, Gogarten S, Shannon P, Morgan M (2014). VariantAnnotation: a Bioconductor package for exploration and annotation of genetic variants. Bioinformatics.

[35] Babraham Bioinformatics - FastQC A Quality Control tool for High Throughput Sequence Data. http://www.bioinformatics.babraham.ac.uk/projects/fastqc/. Accessed 15 Sept 2015.

[36] FASTX-Toolkit. http://hannonlab.cshl.edu/fastx_toolkit/index.html. Accessed 17 Sept 2015.

[37] Ewels P, Magnusson M, Lundin S, Käller M. MultiQC: Summarize analysis results for multiple tools and samples in a single report. Bioinformatics. 2016. doi:10.1093/bioinformatics/btw354. 10.1093/bioinformatics/btw354 PMC5039924 27312411

[38] Afgan E, Baker D, Coraor N, Goto H, Paul IM, Makova KD, Nekrutenko A, Taylor J (2011). Harnessing cloud computing with galaxy cloud. Nat Biotechnol.

[39] Sloggett C, Goonasekera N, Afgan E (2013). BioBlend: automating pipeline analyses within galaxy and CloudMan. Bioinformatics.

[40] D’Antonio M, D’Onorio De Meo P, Pallocca M, Picardi E, D’Erchia AM, Calogero RA, Castrignanò T, Pesole G (2015). RAP: RNA-Seq analysis pipeline, a new cloud-based NGS web application. BMC Genomics.

[41] Torri F, Dinov ID, Zamanyan A, Sam H, Genco A, Petrosyan P, Clark AP, Liu Z, Eggert P, Pierce J, Knowles JA, Ames J, Kesselman C, Toga AW, Potkin SG, Vawter MP, Macciardi F (2012). Next generation sequence analysis and computational genomics using graphical pipeline workflows. Genes.

[42] Rabix W. Reproducible Analyses for Bioinformatics (Rabix). 2015. https://www.rabix.org. Accessed 16 Sept 2015.

[43] WDL W. Workflow Description Language (WDL). 2015. https://github.com/broadinstitute/wdl. Accessed 16 Sept 2015.

[44] Gaidatzis D, Lerch A, Hahne F, Stadler MB (2015). QuasR: quantification and annotation of short reads in R. Bioinformatics.

