# Supplementary material for: systemPipeR: NGS workflow and report generation environment
Source: BMC Bioinformatics. 2016 Sep 20;17:388. doi: 10.1186/s12859-016-1241-0 (PMC5029110; doi:10.1186/s12859-016-1241-0)
Supplement: Additional file 1 — RNA-Seq Workflow Example. Case study to illustrate the utilities of systemPipeR using an RNA-Seq workflow as example. (PDF 89 kb) [file 12859_2016_1241_MOESM1_ESM.pdf]

Additional file 1: RNA-Seq Workflow Example

The following case study illustrates the utilities of *systemPipeR* by guiding the reader through the most important steps of a sample workflow. A typical gene-level RNA-Seq analysis was chosen here because it is currently one of the most widely used applications in the NGS field.

1. *Overview*
   For new users it is particularly convenient to generate new workflow instances with the `genWorkenvir` function provided by the affiliated helper package *systemPipeRdata*. This creates a workflow directory fully populated with sample data and parameter files required for running a chosen workflow template (here RNA-Seq). Custom data can be used by moving them into the pre-generated *data* subdirectory. Most outputs generated by a workflow will be written to a *results* directory at the same level in the directory tree. Next, the user can run the workflow interactively from the R console by executing the commands provided in the corresponding RNA-Seq template script (R/Latex or R/markdown). Entire workflows can be run from start to finish with a single command from the R console using the `Sweave/knitr` or `render` commands or by clicking the corresponding build menu if RStudio is used as working environment. Alternatively, they can be executed from the command-line. In both cases the analysis reports are generated during the workflow run. For new users it is recommended to test workflows first in interactive mode as it is more transparent and it provides more interactive control than the automated batch mode. Importantly, for any workflow with custom data the initial *targets* file is the main input the user needs to provide for running even very complex workflows with multiple steps involving a variety of command-line and/or R-based software tools.
2. *Read preprocessing*
   Read preprocessing routines such as quality and adapter trimming are often optional steps for RNA-Seq experiments. More commonly they are used for other NGS application such as smallRNA-Seq, VAR-Seq and Ribo-Seq, however it is useful to know how to use them for RNA-Seq applications if needed. The `preprocessReads` function allows to apply predefined or custom read preprocessing functions, such as quality filtering or adaptor trimming, to all FASTQ files specified in a *SYSargs* instance. The latter is generated from the corresponding *targets* and *param* files. Internally, the function uses the `FastqStreamer` tool from the *ShortRead* package to stream through large FASTQ files in a memory-efficient manner. The trimmed FASTQ files are written to new files and registered as new inputs in an updated *SYSargs* instance. Parallelization of this and most of the following steps is straightforward with the *BiocParallel* package using either multiple cores on single machines or multiple nodes on computer clusters. Details on parallelization are provided in the vignettes of the *systemPipeR* package.
3. *Read quality reports*
   The `seeFastq/seeFastqPlot` functions generate and plot a series of useful quality statistics for all non-trimmed and/or trimmed FASTQ files specified in the corresponding *SYSargs* instance. This includes per cycle quality box plots, base

proportions, base-level quality trends, relative k-mer diversity, length and occurrence distribution of reads, number of reads above variable quality cutoffs and mean quality distribution.

4. *Alignments*
   Most splice junction aware aligners can be used with *systemPipeR* for aligning RNA-Seq reads to a reference genome, such as *Bowtie2/Tophat2* [1] or *Rsubread* [2]. The corresponding software needs to be installed on a system prior to its use. For instance, to run *Bowtie2/Tophat2* in serial mode on any number of user provided FASTQ files, an appropriate *SYSargs* object is passed on to the `runCommandline` function. For a parallelized run on a computer cluster the same *SYSargs* object is passed on to the `clusterRun` function along with a list object containing the requested amounts of hardware resources such as number of computer nodes, CPU cores and amount of memory. This design greatly simplifies both the usage of command-line software and compute clusters for the user, while also minimizing errors since sample management is automated at this level. For tracking purposes the run parameters of the aligner and their output messages are recored in log files. Subsequently, alignment summary reports are generated with the `alignStats` function which reports the total number of reads available in each input FASTQ file and how many of them aligned to the reference given variable mapping policies. Alignment summary plots can be generated at this step of a workflow as well, including distribution of reads across all features in a genome annotation, and read depth coverage summarized for any number of transcripts (Figures 2C2-3).
5. *Counting reads overlapping exonic gene regions*
   Reads overlapping with annotation ranges of interest can be quantified with different R and command-line tools. The chosen RNA-Seq sample workflow uses the `summarizeOverlaps` function from the *GenomicAlignment* package as default method [3]. `summarizeOverlaps` supports read counting for many feature types and overlap modes such as strand sensitive/insensitive behavior and removal of ambiguous mappings in a manner similar to other read counters, such as `featureCounts` from the *rsubread* package [2]. Typically, for gene-level expression profiling the user usually wants to perform read counting on exonic gene regions while ignoring reads mapping to regions where several genes overlap. The resulting read counts represent the raw gene expression data of an RNA-Seq experiment. These are stored in a *SummarizedExperiment* object which can be exported to a *data.frame* or a tabular file. The rows and columns of these objects usually represent gene and sample identifiers, respectively. To evaluate similarities among samples and/or biological replicates in RNA-Seq experiments, the read counts can be used to generate sample similarity trees or scatter plots using hierarchical clustering or multidimensional scaling (MDS), respectively (Figure 2C5).
6. *DEG analysis*
   The analysis of DEGs can be performed in *systemPipeR* with the widely used *edgeR* or *DESeq2* packages. The helper functions `run_edgeR` and `run_DESeq2` are provided to perform simple pair-wise comparisons. These convenience functions perform the DEG analysis in batch mode for any number of user defined sample

comparisons. If sample comparisons are provided in the header section of the initial *targets* file, the DEG analysis can be fully automated. For more complex experimental designs, users can run the DEG analysis methods as instructed in the *edgeR* and *DESeq2* vignettes. The results of the DEG analysis can be queried and plotted with a filter function (Figure 2C4). Filtering instances are stored as a list, where gene identifiers meeting the filter criteria (*e.g.* below certain false discovery rate, FDR) are stored as character vectors. Both the raw DEG results (e.g. FDRs) and filter list instances can be directly passed on to downstream gene set enrichment routines.

7. *Gene set enrichment analysis*
   Enrichment analysis of Gene Ontology (GO) terms can be performed with many Bioconductor packages addressing this need. *systemPipeR* provides convenience functions to automate this analysis for large numbers of gene sets and to generate tabular report files. For instance, the gene sets obtained from the DEG analysis can be directly used for GO term enrichment analysis in batch mode using the `GOCluster_Report` function. This performs GO term enrichment analysis based on the hypergeometric distribution and returns the corresponding raw and adjusted p-values corrected for multiple testing. Subsequently, the results can be visualized with the `goBarplot` function.
8. *Workflow report*
   The results of the above workflow steps are summarized in a detailed analysis report. If required, users can add custom content to the reports describing experimental conditions as well as other useful details. By default, summary tables and plots generated during workflow runs are shown in the reports directly, while links to result files are provided for large data sets, such as short read alignments, gene-level read counts and complete DEG results. Also included are code boxes containing the exact code used for running each step in a workflow. Software and library versions used by a workflow are recorded at the end of a report. When a workflow is repeated entirely or in parts then the corresponding report sections will be updated automatically. Users can also customize workflow steps and/or add new ones as needed. In summary, the workflow reporting infrastructure in *systemPipeR* provides all relevant details required to reproduce complex NGS analyses.

References

1. Kim, D., Pertea, G., Trapnell, C., Harold, P., Kelley, R., Salzberg, S.L.: TopHat2: accurate alignment of transcriptomes in the presence of insertions, deletions and gene fusions. Genome Biol. **14**(4) (2013)
2. Liao, Y., Smyth, G.K., Shi, W.: The Subread aligner: fast, accurate and scalable read mapping by seed-and-vote. Nucleic Acids Res. **41**(10), 108 (2013)
3. Lawrence, M., Huber, W., Pagès, H.: Software for computing and annotating genomic ranges
